# Biobased Polymers via Radical Homopolymerization and Copolymerization of a Series of Terpenoid-Derived Conjugated Dienes with *exo*-Methylene and 6-Membered Ring

**DOI:** 10.3390/molecules25245890

**Published:** 2020-12-12

**Authors:** Takenori Nishida, Kotaro Satoh, Masami Kamigaito

**Affiliations:** 1 Department of Molecular and Macromolecular Chemistry, Graduate School of Engineering, Nagoya University, Furo-cho, Chikusa-ku, Nagoya 464-8603, Japan; nishida@chiral.apchem.nagoya-u.ac.jp (T.N.); satoh@cap.mac.titech.ac.jp (K.S.); 2 Department of Chemical Science and Engineering, Tokyo Institute of Technology, School of Materials and Chemical Technology, 2-12-1-H120 Ookayama, Meguro-ku, Tokyo 152-8550, Japan

**Keywords:** terpenoid, *exo*-methylene, conjugated diene, renewable monomer, biobased polymer, radical polymerization, copolymerization, living radical polymerization, RAFT polymerization, heat-resistant polymer

## Abstract

A series of *exo*-methylene 6-membered ring conjugated dienes, which are directly or indirectly obtained from terpenoids, such as β-phellandrene, carvone, piperitone, and verbenone, were radically polymerized. Although their radical homopolymerizations were very slow, radical copolymerizations proceeded well with various common vinyl monomers, such as methyl acrylate (MA), acrylonitrile (AN), methyl methacrylate (MMA), and styrene (St), resulting in copolymers with comparable incorporation ratios of bio-based cyclic conjugated monomer units ranging from 40 to 60 mol% at a 1:1 feed ratio. The monomer reactivity ratios when using AN as a comonomer were close to 0, whereas those with St were approximately 0.5 to 1, indicating that these diene monomers can be considered electron-rich monomers. Reversible addition fragmentation chain-transfer (RAFT) copolymerizations with MA, AN, MMA, and St were all successful when using *S*-cumyl-*S*’-butyl trithiocarbonate (CBTC) as the RAFT agent resulting in copolymers with controlled molecular weights. The copolymers obtained with AN, MMA, or St showed glass transition temperatures (*T*_g_) similar to those of common vinyl polymers (*T*_g_ ~ 100 °C), indicating that biobased cyclic structures were successfully incorporated into commodity polymers without losing good thermal properties.

## 1. Introduction

Polymeric materials, such as plastics, rubbers, and fibers, are indispensable products in our daily life and are produced from a variety of low-molecular-weight molecules as building blocks. Radical polymerization is one of the most effective and widely used techniques to produce a wide variety of polymeric materials from various vinyl monomers due to the high reactivity of radical species and its robustness toward polar functional groups in the monomer substituents as well as aqueous compounds in the reaction mixture [1]. In addition, recent progress in controlled/living or reversible deactivation radical polymerization (RDRP) has dramatically expanded the scope of radical polymerization to synthesize precision polymers with various well-defined structures that can be used as high-performance materials [1,2,3,4,5,6,7,8,9,10].

Sustainable industrial development is now indispensable in view of environmental issues, such as global warming, depletion of fossil resources, and plastic waste. One of the promising solutions to these issues is to use renewable resources, in particular plant-derived molecules, as an alternative to the petroleum-derived monomers for synthetic polymers [11,12,13,14,15,16,17,18,19,20,21]. Moreover, the unique structures of naturally occurring molecules can bestow biobased synthetic polymers with characteristic features that are difficult to attain using simpler petroleum-derived molecules, resulting in novel high-performance materials.

A number of vinyl compounds are produced in nature and are found in a family of abundant natural compounds, such as terpenoids and phenylpropanoids [22,23,24,25]. As we have proposed, these biobased vinyl compounds can be classified into similar vinyl monomer families, such as nonpolar olefins, styrenes, and acrylic monomers, to those of petroleum-derived monomers, depending on their substituents and structures [26,27,28,29]. For example, renewable polar monomers with conjugated carbonyl groups, such as tulipalin A and itaconic acid, are considered to be acrylic monomers [30,31,32,33,34,35,36,37,38,39,40,41], whereas phenylpropanoids and their derivatives, such as anethole and vinylguaiacol, are considered to be styrenes [42,43,44,45,46,47,48]. These conjugated monomers are generally reactive in radical polymerizations due to the C=C bonds conjugated with carbonyl or phenyl groups, resulting in relatively stable propagating radical species.

In contrast, among various terpenes [22,23,24,25], the most abundant terpenes are pinene and limonene, which are unconjugated nonpolar olefins without carbonyl or aromatic groups and have difficulty in undergoing radical homopolymerization [49,50,51,52,53,54,55]. However, these unconjugated terpenes are radically copolymerized with electron-deficient polar monomers, such as acrylates and maleimides, resulting in biobased copolymers with a relatively high terpene incorporation ratio of up to 50 mol%. In contrast, a linear conjugated diene, such as myrcene, is another nonpolar olefin terpene and is radically homopolymerized, resulting in rubbery materials [56,57,58,59,60,61,62]. However, linear conjugated dienes undergo concurrent Diels-Alder reactions with electron-deficient vinyl monomers via their *s*-*cis* forms during radical copolymerization.

**Scheme 1:**
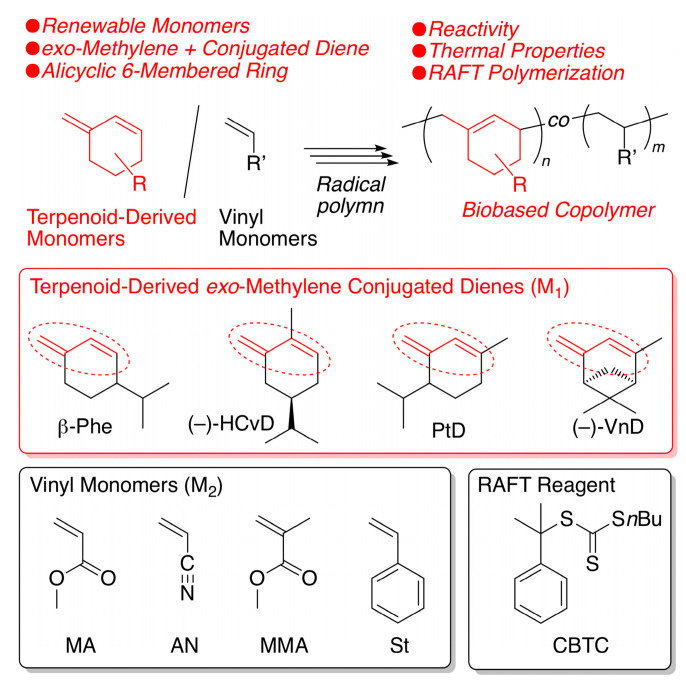
Radical homo- and copolymerization of a series of terpenoid-derived *exo*-methylene 6-membered-ring conjugated dienes.

Another nonpolar conjugated diene observed in terpenoids is a *transoid* cyclic conjugated diene with a reactive *exo*-methylene group, such as β-phellandrene (β-Phe) (Scheme 1). As we briefly reported in a patent [63], β-Phe possesses high reactivity in cationic polymerization resulting in heat-resistant cycloolefin polymers after subsequent hydrogenation. More recently, we synthesized another *exo*-methylene 6-membered ring conjugated diene (HCvD: hydrogenated carvone-derived diene) from carvone, which is an abundant naturally occurring α,β-unsaturated carbonyl compound, via simple chemical transformations consisting of hydrogenation and Wittig reactions [64]. We have also found that HCvD is readily polymerized in cationic polymerization, resulting in alicyclic hydrocarbon polymers with good thermal properties. In addition to HCvD, other *exo*-methylene *transoid* 6-membered ring conjugated dienes (PtD and VnD) have been prepared from a series of naturally occurring α,β-unsaturated carbonyl compounds, i.e., piperitone and verbenone, respectively, via the Wittig reaction [65].

The *exo*-methylene-conjugated dienes can be radically homo- and copolymerized with petroleum-derived common vinyl monomers. However, there have been no comprehensive studies on radical homo- and copolymerization of these terpenoid-derived *exo*-methylene 6-membered ring conjugated dienes except for a few results on the homo- and copolymerizations with some specific monomers, such as styrene, acrylonitrile, and *N*-phenylmaleimide [65,66,67]. These results suggest that some of the *exo*-methylene 6-membered ring conjugated dienes do not seem highly reactive in radical homopolymerization and that they can be copolymerized with electron-deficient monomers. Similar results were reported for 3-methylenecyclopentene, which is an *exo*-methylene 5-membered ring conjugated diene obtained from the ring-closing metathesis of myrcene [68,69].

In this work, we fully examined radical homo- and copolymerizations of a series of terpenoid-derived *exo*-methylene 6-membered ring conjugated dienes (β-Phe, HCvD, PtD, and VnD) using various common vinyl monomers, including methyl acrylate (MA), acrylonitrile (AN), methyl methacrylate (MMA), and styrene (St), as comonomers to evaluate their reactivity in racial polymerization and the thermal properties of the resulting biobased polymers as heat-resistant materials with unique alicyclic skeletons originating from naturally occurring terpenoid molecules. 

## 2. Results and Discussion

### 2.1. Radical Homopolymerization

Radical homopolymerizations of a series of terpenoid-derived *exo*-methylene 6-memered ring conjugated dienes, β-Phe, (−)-HCvD, PtD, and (−)-VnD, which were obtained as previously reported [63,64], were examined in toluene at 60 and 100 °C using 2,2′-azobisisobutyronitirle (AIBN) and 2,2′-azobis(*N*-butyl-2-methylpropionamide) (VAm-110), respectively, as radical initiators (Table 1). The polymerizations of all monomers at 60 °C were totally sluggish, resulting in a trace amount of polymers with number-average molecular weights (*M*_n_) less than 10^4^. Upon increasing the temperature to 100 °C, although the polymerizations were still slow, they visibly proceeded to result in soluble polymers with relatively high molecular weights (*M*_n_ > 10^4^) except for (–)-VnD, which resulted in only low molecular weight oligomers (*M*_n_ < 10^3^) at 60 and 100 °C. All size-exclusion chromatography (SEC) curves of the obtained polymers showed unimodal shapes (Figure S1), suggesting that cross-linking reactions with the unconjugated olefins formed in the polymer chains rarely occurred. 

**Figure 1:**
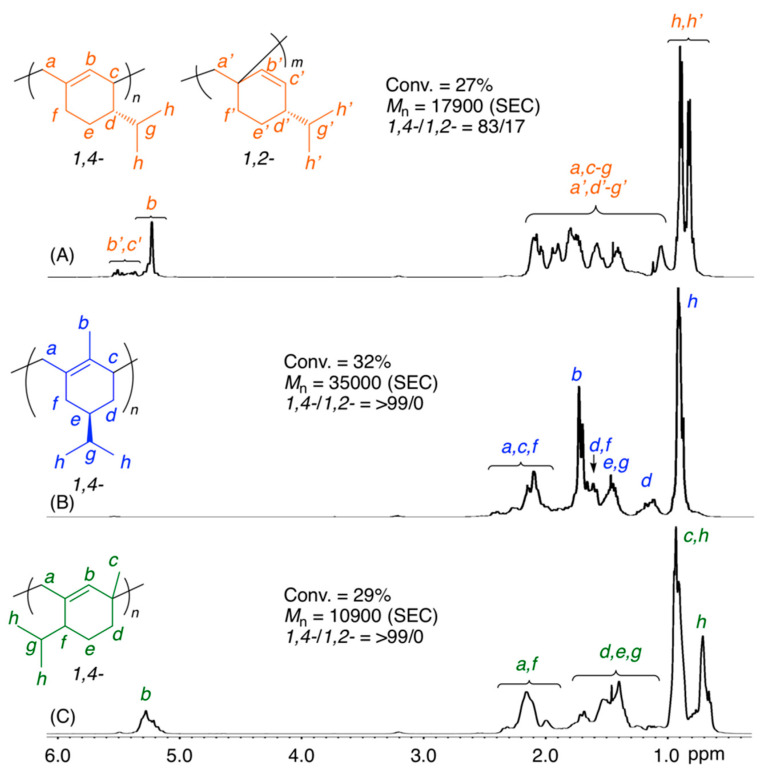
^1^H NMR spectra (in CDCl_3_ at 55 °C) of poly(β-Phe) (A), poly((–)-HCvD) (B), and poly(PtD) (C) obtained in the radical polymerization of the *exo*-methylene conjugated dienes: [M]_0_/[VAm-110]_0_ = 5000/30 mM in toluene at 100 °C.

The ^1^H NMR spectra of the obtained polymers showed that the polymerization of (−)-HCvD and PtD proceeded via regioselective 1,4-conjugated addition (Figure 1). There are no olefinic protons at approximately 5–6 ppm for poly((−)-HCvD), whereas no methyl groups attached to olefins were observed at approximately 1.5–2.0 ppm for poly(PtD). Furthermore, for poly(PtD), the peak intensity ratios of methyl protons (0.6–1.0 ppm), which are attached to saturated carbons, to olefin (5.1–5.3 ppm) protons were 8.9:1, indicating not only regioselective 1,4-conjugated addition but also no cross-linking reactions. However, the 1,4-regioselectivity was not complete for poly(β-Phe), which consisted of 83/17 of 1,4-/1,2-units as estimated from the peak intensity ratios of the olefinic to alkyl protons, in contrast to the complete 1,4-regioselective polymerization of β-Phe via the cationic mechanism as briefly reported previously [63]. 

The stereoregularity is another interesting issue of the obtained polymers. The poly((−)-HCvD) obtained via radical polymerization of the chiral monomer was easily soluble in common organic solvents, such as hexane and tetrahydrofuran, whereas an insoluble polymer in these solvents was obtained via the regioselective and stereospecific cationic polymerization of the same chiral monomer [64]. The ^13^C NMR spectrum of poly((−)-HCvD) obtained via the radical process showed a larger number of peaks than that obtained in the cationic process, in which the chiral center attached to the isopropyl group can dictate the stereospecificity at low temperature (Figure S2).

Thus, both regioselectivity and stereospecificity in the polymerizations of a series of terpenoid-derived *exo*-methylene conjugated dienes were influenced by the propagating species, among which the radical species resulted in lower selectivities due to the free radical propagating species as well as the higher reaction temperature.

The thermal properties of poly(β-Phe), poly((−)-HCvD), and poly(PtD) obtained in radical polymerization at 100 °C were evaluated by differential scanning calorimetry (DSC) (Figure S3). The glass transition temperatures (*T*_g_) were observed for all polymers. The *T*_g_s of poly((−)-HCvD) and poly(PtD) were approximately 110 °C due to the cyclic structures incorporated into the main chain via regioselective 1,4-conjugation, whereas the *T*_g_ of poly(β-Phe) was 66 °C, which was lower than that of poly(β-Phe) (*T*_g_ = 87 °C) obtained in cationic polymerization [63] due to the decreased 1,4-regioselectivity in radical polymerization. In addition, the poly((−)-HCvD) obtained in radical polymerization showed a slightly lower *T*_g_ than that obtained in the cationic polymerization of (−)-HCvD (*T*_g_ = 105 vs. 114 °C) and showed no melting peak due to the lack of stereospecificity in radical polymerization [64].

These results show that a series of the *exo*-methylene 6-membered ring conjugated dienes are slowly homopolymerized by radical species and that the regioselectivity and stereospecificity are lower for some monomers than those in cationic polymerization.

### 2.2. Radical Copolymerization with Various Common Vinyl Monomers

A series of terpenoid-derived *exo*-methylene 6-membered ring conjugated dienes were then copolymerized with various common vinyl monomers, such as MA, AN, MMA, and St, at a 1:1 feed ratio in toluene at 60 °C using AIBN (Figure S4 and Table S1). Radical copolymerizations occurred in almost all cases except for copolymerization with (−)-VnD, in which conversions of both (−)-VnD and comonomers were very low and the obtained products were only oligomers (*M*_n_ ≤ 1 × 10^3^). Therefore, (−)-VnD is only slightly reactive in radical homo- and copolymerization, probably because the bulky structure with a bicyclic structure and a methyl group at the 4-position makes propagation very difficult in the radical process. Although the other *exo*-methylene-conjugated dienes were all copolymerized, the reactions at 60 °C were generally slow and resulted in polymers with relatively low molecular weights (*M*_n_ = 1 × 10^3^ – 2 × 10^4^). 

**Figure 2:**
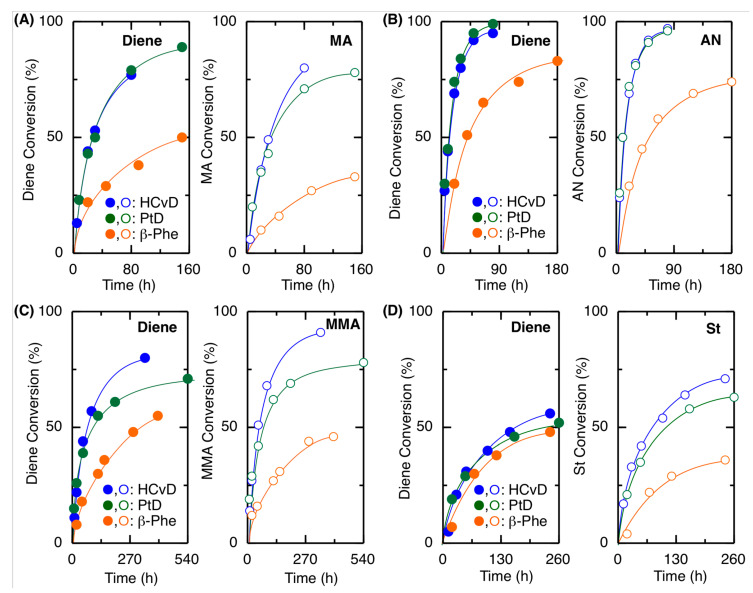
Time-conversion curves for the radical copolymerization of *exo*-methylene 6-memered-ring conjugated dienes with MA (**A**), AN (**B**), MMA (**C**), or St (**D**) as a comonomer: [diene]_0_/[comonomer]_0_/[VAm-110]_0_ = 1500/1500/30 in toluene at 100 °C.

Therefore, the radical copolymerizations of β-Phe, (–)-HCvD, and PtD with MA, AN, MMA, and St at a 1:1 feed ratio were investigated at 100 °C in toluene using VAm-110 as the radical initiator (Table 2 and Figure 2 and Figure S5). The polymerization rates, monomer conversion, and molecular weight of the resulting copolymers (*M*_n_ = 6 × 10^3^ – 4 × 10^4^) were drastically improved, indicating that a higher reaction temperature is favorable for not only homo- but also copolymerization of a series of the *exo*-methylene 6-membered ring conjugated dienes. 

In general, electron-deficient comonomers were suitable for copolymerization, where the reaction rates, conversions, and molecular weights increased in the following order: AN > MA > MMA > St, suggesting that these *exo*-methylene cyclic conjugated dienes are electron-rich monomers. Among the cyclic conjugated dienes, (−)-HCvD and PtD showed similar reactivities, resulting in polymers with similar molecular weights in all cases, while β-Phe resulted in slower polymerizations and lower molecular weights. However, the incorporation ratio of β-Phe was generally greater than that of (−)-HCvD and PtD for any comonomer (Table 2). These results suggest that relatively high incorporation of β-Phe leads to a high probability of the β-Phe-derived propagating radical species, by which the propagation is slow to result in copolymers with relatively low molecular weights. More details on the monomer reactivity ratio will be studied and discussed in the next section.

**Figure 3:**
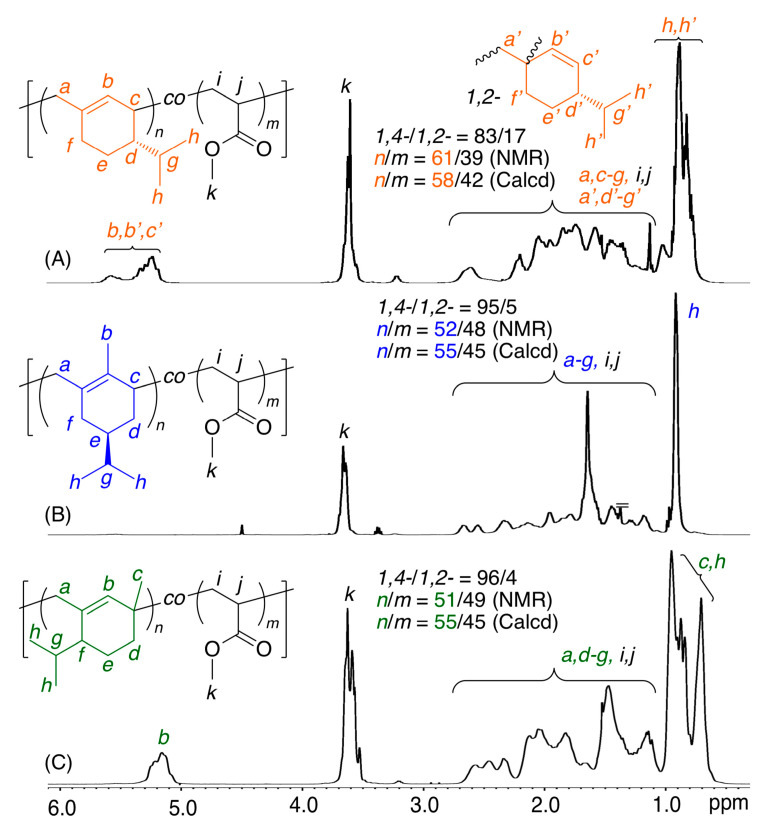
^1^H NMR spectra (in CDCl_3_ at 55 °C) of copolymers obtained in the radical copolymerization of β-Phe (**A**), (−)-HCvD (**B**), or PtD (**C**) with MA: [diene]_0_/[MA]_0_/[VAm-110]_0_ = 1500/1500/30 mM in toluene at 100 °C.

All the obtained copolymers were analyzed by ^1^H NMR to clarify the copolymer structures, particularly regarding the incorporation ratio of comonomers and the regiospecificity of the diene monomers during the copolymerizations (Figure 3, Figures S6–S8). For example, Figure 3 shows spectra of a series of copolymers obtained with β-Phe, (−)-HCvD, or PtD and MA. In addition to the characteristic signals of the conjugated diene units, all the spectra showed additional signals of MA units, including not only main-chain methylene and methine protons but also methyl ester protons (*k*). In particular, olefinic protons (*b*) were observed at approximately 5–6 ppm for the copolymers of β-Phe or PtD (Figure 3A,C), although the peaks were slightly broader than those of the homopolymers of β-Phe or PtD (Figure 1A,C). In addition, almost no olefinic protons were observed for the copolymers of (−)-HCvD (Figure 3B), indicating that the high 1,4-conjugated addition polymerization also proceeded in the copolymerization. For the other various copolymers with AN, MMA, or St (Figures S6–S8), β-Phe or PtD olefinic protons were similarly observed at approximately 4–6 ppm, although the chemical shifts were dependent on the comonomers. Furthermore, for the copolymers of (−)-HCvD with all comonomers, almost no olefinic protons were observed.

The incorporation ratios of the conjugated dienes (M_1_) and common vinyl monomers (M_2_) were calculated by the peak intensity ratios of the diene and vinyl monomer units in the ^1^H NMR spectra. The incorporation ratios (M_1_/M_2_(NMR)) obtained by NMR of the resulting copolymers were close to the calculated values (M_1_/M_2_(Calcd)) based on the feed ratio and monomer conversions, indicating that the consumed monomers were efficiently incorporated into the copolymers. Furthermore, the regioselectivities of the diene units were calculated by the olefinic protons and other protons for all copolymers. The regioselectivities in the copolymers were basically the same as those for the homopolymers. Namely, high 1,4-selectivity was attained for (−)-HCvD and PtD, whereas the 1,4-selectivity for β-Phe was approximately 80%. 

Thus, a series of terpenoid-derived *exo*-methylene 6-membered ring conjugated dienes are efficiently copolymerized with various common vinyl monomers, including MA, AN, MMA, and St, via mainly 1,4-conjugated additions resulting in copolymers with biobased monomer incorporation ratios ranging from 40 to 60 mol% at a 1:1 feed ratio. 

### 2.3. Monomer Reactivity Ratio

**Figure 4:**
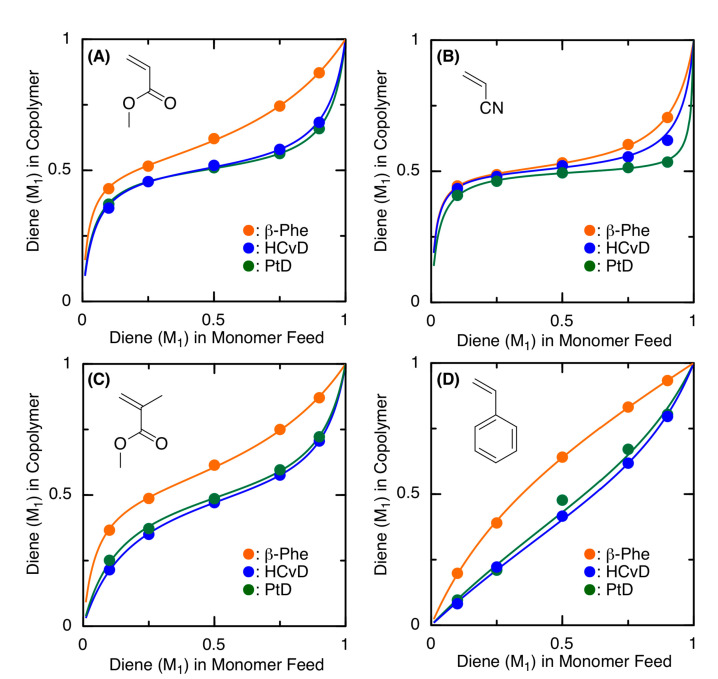
Copolymer composition curves for the radical copolymerization of terpenoid-derived *exo*-methylene 6-membered-ring conjugated dienes as M_1_ and MA (**A**), AN (**B**), MMA (**C**), or St (**D**) as M_2_ with VAm-110 obtained at varying monomer feed ratios ([M_1_]_0_/[M_2_]_0_ = 1/9, 1/3, 1/1, 3/1, 9/1): ([M_1_]_0_ + [M_2_]_0_)/[VAm-110]_0_ = 5000/30 mM in toluene at 100 °C. The solid lines were fitted by the Kelen–Tüdõs method for the terminal model (see *r*_1_ and *r*_2_ values in Table 2).

To further clarify the copolymerizability of a series of the *exo*-methylene 6-membered ring conjugated dienes in radical copolymerizations, the monomer reactivity ratios were determined by analyzing the comonomer compositions of the copolymers obtained in the initial stages (total conversion < 10%) of the copolymerizations at various monomer feed ratios ([M_1_]_0_ + [M_2_]_0_ = 5000 mM, [VAm-110]_0_ = 30 mM in toluene at 100 °C). Figure 4 shows the copolymer composition curves, in which the diene unit content in the resulting copolymers was plotted against the diene content in the monomer feeds. The data were analyzed using the terminal model and were fitted well with the solid lines obtained using the Kelen–Tüdõs method. The obtained *r*_1_ and *r*_2_ values are summarized in Table 2. 

In general, all the plots and fitted curves for HCvD and PtD were close to each other, whereas those for β-Phe were located above the others, indicating that the incorporation of β-Phe units in the resulting copolymers was consistently greater than that of HCvD and PtD at the same feeds. Correspondingly, the *r*_1_ values of β-Phe were consistently larger than those of HCvD and PtD. In addition, the *r*_1_ values of HCvD and PtD were relatively close. These results indicate that HCvD and PtD, which both undergo selective 1,4-regioselective propagation, possess similar reactivities, while β-Phe with a decreased 1,4-regioselectivity shows a higher copolymerizability than HCvD and PtD.

When focusing on the copolymerizations with electron-deficient comonomers (MA, AN, and MMA), both *r*_1_ and *r*_2_ were less than 1, and in particular, those for HCvD and PtD with AN were less than 0.1, indicating that alternating copolymerization predominantly occurs, as also suggested by the almost constant plot for the various monomer feed ratios (Figure 4B). This is because the *exo*-methylene-conjugated diene can be considered an electron-rich monomer, whereas AN is the most electron-deficient monomer among the comonomers. For the copolymerizations with St, the sequence of the copolymer is more random because both of the *r* values are relatively close to 1, although both monomers are slightly more reactive than the other monomers. 

When the *r* values of the *exo*-methylene cyclic conjugated dienes are compared with those of a linear conjugated diene, such as butadiene (Bd), they are partially different. The *r*_1_ values, using Bd as M_1_ and the other common vinyl monomers as M_2_, are 1.09 (MA), 0.36 (AN), 0.75 (MMA), and 1.44 (St) [70], which are greater than those of the *exo*-methylene cyclic conjugated dienes due to the low homopolymerizability of the *exo*-methylene cyclic dienes most likely caused by the steric hindrance around the *exo*-methylene group. In contrast, the *r*_2_ values for comonomers M_2_ with Bd as M_1_ are 0.07 (MA), 0.05 (AN), 0.25 (MMA), and 0.84 (St) [70], which are similar to those obtained in copolymerizations with the *exo*-methylene cyclic conjugated dienes, indicating that the propagating radical species derived from common comonomers can react with cyclic conjugated dienes with reactivity similar to that of the Bd monomer.

Thus, a series of terpenoid-derived *exo*-methylene 6-membered ring conjugated dienes possess sufficiently high copolymerizability with various common vinyl monomers to be efficiently incorporated into the polymer main chains via 1,4-conjugated additions, which results in cyclic structures in the main chain of the copolymers. 

### 2.4. Thermal Properties of Copolymers

**Figure 5:**
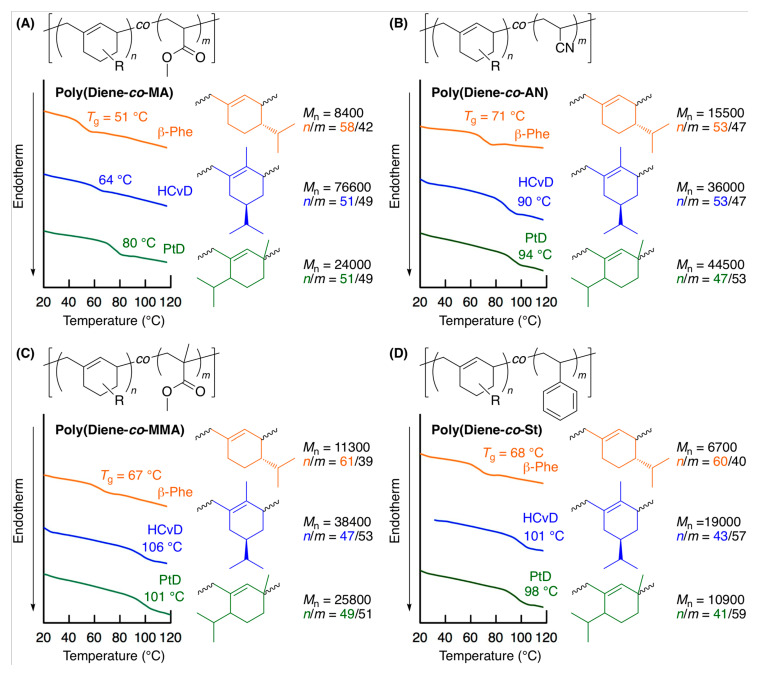
Differential scanning calorimetry (DSC) curves of copolymers obtained in the radical copolymerization of terpenoid-derived *exo*-methylene 6-membered ring conjugated dienes with MA (**A**), AN (**B**), MMA (**C**), and St (**D**).

The thermal properties of the various obtained copolymers were evaluated by DSC (Figure 5). All the obtained copolymers showed their characteristic *T*_g_s. For the copolymers of MA, the *T*_g_ values were much higher than that of the homopolymer of MA (*T*_g_ ~ 10 °C) [70] due to the incorporated terpenoid-derived alicyclic structure, and the *T*_g_ values ranged from 50 to 80 °C depending on the structure of the cyclic conjugated dienes. Furthermore, the *T*_g_ values of the copolymers of MMA, AN, and St with (−)-HCvD and PtD were approximately 100 °C, which is similar to the *T*_g_ values of the homopolymers, indicating that the biobased units can be introduced into common vinyl polymers without losing their good thermal properties. This could be a unique benefit of the biobased *exo*-methylene 6-membered ring conjugated dienes as comonomers for MMA, AN, and St, in contrast to linear olefins and dienes, which generally have lower *T*_g_s due to the soft hydrocarbon units. Thus, terpenoid-derived alicyclic *exo*-methylene conjugated dienes are promising monomers as novel rigid hydrocarbon units that can be introduced at a high incorporation ratio in radical copolymerization. 

### 2.5. Reversible Addition Fragmentation Chain-Transfer (RAFT) Copolymerization of (–)-HCvD and Various Common Vinyl Monomers

**Figure 6:**
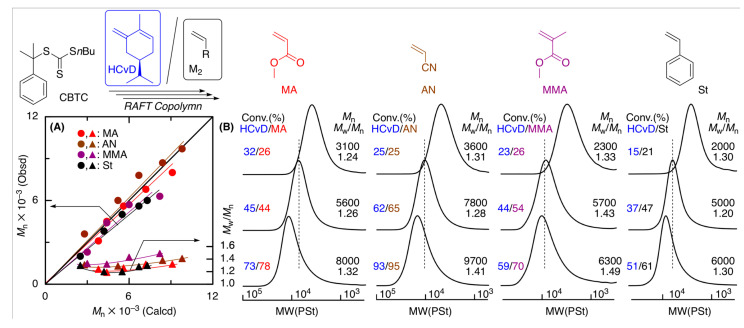
(**A**) *M*_n_, *M*_w_/*M*_n_, and (**B**) SEC curves of the copolymers obtained in the RAFT copolymerization of (–)-HCvD with various common vinyl monomers (M_2_): [(−)-HCvD]_0_/[M_2_]_0_/[CBTC]_0_/[VAm-110]_0_ = 1500/1500/30/10 mM in toluene at 100 °C.

To control the copolymerization for precision synthesis of the novel biobased polymers, the RAFT copolymerization of (−)-HCvD and various common vinyl monomers (MA, MMA, AN, and St) was investigated at a 1:1 feed ratio using *S*-cumyl-*S’*-butyl trithiocarbonate (CBTC) as the RAFT agent and VAm-110 as the radical initiator in toluene at 100 °C (Figure 6 and Figure S9). In all cases, the copolymerizations proceeded smoothly even in the presence of the RAFT agent at conversion ratios similar to those in radical copolymerization without the RAFT agent (Figure S9).

The *M*_n_ values of the resulting copolymers increased in direct proportion to total monomer conversion in all cases and were close to the calculated values assuming that one molecule of CBTC generates one polymer chain (Figure 6A). In addition, as the polymerization proceeded, the SEC curves shifted to higher molecular weights while maintaining relatively narrow molecular weight distributions (*M*_w_/*M*_n_ = 1.2–1.5) (Figure 6B).

The ^1^H NMR spectra (Figure S10) of the copolymers obtained in the RAFT copolymerization showed similar large absorptions originating from the terpenoid-based cyclic conjugated dienes and common vinyl monomer units and small signals of both the α- and ω-terminals derived from the RAFT agent, for which signals at 7.3–7.4 and 3.3–3.4 ppm can be attributed to the aromatic and methylene protons originating from CBTC, respectively. The incorporations of (−)-HCvD units into the copolymers were similarly calculated by the peak intensity ratios and were almost the same as those for the copolymers obtained in radical copolymerization without the RAFT agent. The *M*_n_ values calculated from the peak intensity ratios of the α- and ω-RAFT terminals to the repeating units were close to both *M*_n_(SEC) and *M*_n_(Calcd), suggesting that one CBTC molecule generates one polymer chain. These results indicate that CBTC is suitable for controlling the RAFT copolymerization of (−)-HCvD and various common vinyl monomers to produce copolymers with controlled molecular weights and chain-end groups, which can be used for synthesis of the novel biobased functional polymers.

## 3. Materials and Methods 

### 3.1. Materials

Toluene (KANTO, >99.5%; H_2_O < 10 ppm) and tetrahydrofuran (THF) (KANTO, >99.5%; H_2_O < 0.001%) was dried and deoxygenized by passage through Glass Contour Solvent Systems columns before use. (Methyl)triphenylphosphonium bromide (KANTO, >98%) and potassium *tert*-butoxide (Tokyo Chemical Industry, >97%) were used as received. Piperitone (mixture of enantiomers, predominantly (*R*)-form, Tokyo Chemical Industry, >94%), angelica seed oil (Aldrich, containing approximately 80% β-phellandrene), methyl acrylate (MA) (Tokyo Chemical Industry, >99%), acrylonitrile (AN) (Tokyo Chemical Industry, >99%), methyl methacrylate (MMA) (Tokyo Chemical Industry, >98%), styrene (St) (Kishida, 99.5%), and 1,2,3,4-tetrahydronaphthalene (Wako, 97%) were distilled under reduced pressure before use. (−)-Verbenone (Tokyo Chemical Industry, >95%) was purified by column chromatography on silica gel with *n*-hexane/ethyl acetate (6/4) as the eluent to result in the pure compound (>99%). *N*-Cyclohexylmaleimide (Aldrich, 97%) was purified by recrystallization from toluene. 2,2′-Azobisisobutyronitirle (AIBN) (Kishida, >99%) and 2,2′-azobis(*N*-butyl-2-methylpropionamide) (VAm-110) (Wako, >95%) were purified by recrystallization from methanol and hexane. (−)-HCvD [63] and *S*-cumyl-*S*’-butyl trithiocarbonate (CBTC) [71] were synthesized according to the literature.

### 3.2. Purification of β-Phellandrene (β-Phe)

β-Phellandrene (β-Phe) was obtained from angelica seed oil. *N*-Cyclohexylmaleimide (24.3 g, 0.14 mol) was added to distilled angelica seed oil (75.9 g), which contained β-Phe (92%) and a small amount of linear and *cisoid*-conjugated dienes, such as myrcene and α-phellandrene as impurities, and the linear and *cisoid* compounds were removed using the Diels-Alder reaction. The reaction mixture was stirred at 90 °C for 1 h. After cooling the reaction mixture to 0 °C for quenching, the crude product was purified by column chromatography on silica gel with *n*-hexane as the eluent to yield β-Phe with a higher purity (27 g, 35% yield, 99% purity). Further purification was conducted by distillation to produce a colorless β-Phe oil (14 g, 19% yield, >99% purity, bp 65 °C/2700 Pa. [α]_D_^25^ = +27°).

### 3.3. Synthesis of (–)-VnD

(−)-VnD was prepared by the Wittig reaction of purified (−)-verbenone. (Methyl)triphenylphosphonium bromide (140.2 g, 0.392 mol) was suspended in dry THF (700 mL) at 0 °C in a three-necked 2 L round-bottom flask equipped with three-way stopcocks. Potassium *tert*-butoxide (44.1 g, 0.392 mol) was added resulting in a yellow suspension. After 30 min, (−)-verbenone (40 mL, 0.261 mol) was added to the yellow suspension resulting in an orange product, and the reaction mixture was stirred at room temperature. After 16 h, the conversion of verbenone reached >99% by ^1^H NMR. Then, water (400 mL) was added to quench the reaction. The organic layer was concentrated by rotary evaporation. Hexane (300 mL) was added to the obtained residue. After the mixture was filtered to remove the precipitated triphenylphosphine oxide, the obtained solution was concentrated. The obtained residue was similarly treated again to yield the crude (−)-VnD product (32.1 g, 82% yield). Further purification was conducted by distillation to produce a colorless oil of pure (−)-VnD (28.9 g, 74% yield, >99% purity, bp 55 °C/1600 Pa, [α]_D_^25^ = −44°). ^1^H NMR (CDCl_3_, r.t.): δ 0.82 (s, 3H, C*H*_3_), 1.35 (s, 3H, C*H*_3_), 1.43-1,46 (d, 1H, C*H*_2_), 1.78 (s, 3H, CH_2_=C-CH=C-C*H*_3_), 2.09–2.12 (t, 1H, CH_2_=C-CH=C-C*H*), 2.48–2.53 (m, 1H, C*H*_2_), 2.58–2.61 (s, 1H, CH_2_=C(C*H*)-CH=C), 4.56 (s, 2H, C*H*_2_=C-CH=C), 5.77 (s, 1H, CH_2_=C-C*H*=C). ^13^C NMR (CDCl_3_, r.t.): δ 21.9 (*C*H_3_), 22.9 (CH_2_=C-CH=C-*C*H_3_), 26.3 (*C*H_3_), 35.5 (*C*H_2_), 43.6 (*C*(CH_3_)_2_), 48.3 (CH_2_=C-CH=C-*C*H), 51.4 (CH_2_=C(*C*H)-CH=C), 104.9 (*C*H_2_=C-CH=C), 121.0 (CH_2_=C-*C*H=C), 147.8 (CH_2_=C-CH=*C*), 149.7 (CH_2_=*C*-CH=C). ^1^H, ^13^C, COSY, and HMQC NMR spectra are shown in Figures S11–S13.

### 3.4. Synthesis of PtD

PtD was prepared by the Wittig reaction of distilled piperitone. (Methyl)triphenylphosphonium bromide (131.6 g, 0.369 mol) was suspended in dry THF (700 mL) at 0 °C in a three-necked 2 L round-bottom flask equipped with three-way stopcocks. Potassium *tert*-butoxide (41.3 g, 0.369 mol) was added to the suspension resulting in a yellow suspension. After 30 min, piperitone (40 mL, 0.246 mol) was added to the yellow suspension to produce an orange product, and the reaction mixture was stirred at room temperature. After 16 h, the conversion of piperitone reached >99% by ^1^H NMR. Then, water (400 mL) was added to quench the reaction. The organic layer was concentrated by rotary evaporation. Hexane (300 mL) was added to the obtained residue. After the mixture was filtered to remove the precipitated triphenylphosphine oxide, the obtained solution was concentrated. The crude product after the Wittig reaction (30.3 g, 82% yield, 81% purity) was purified by column chromatography on silica gel with *n*-hexane as the eluent to yield the product with a higher purity (19.9 g, 36% yield, 94% purity). Further purification was conducted by distillation to produce the colorless PtD oil (16.1 g, 29% yield, 95% purity, bp 70 °C/1600 Pa, [α]_D_^25^ = +16°). ^1^H NMR (CDCl_3_, r.t.): δ 0.88–0.92 (dd, 6H, cy-CH(C*H*_3_)_2_), 1.62–1.71 (m, 2H, CH(*i*Pr)-C*H*_2_-CH_2_, cy-C*H*(CH_3_)_2_), 1.73 (s, 3H, CH_2_=C-CH=C-C*H*_3_), 1.78–1.94 (m, 3H, CH(*i*Pr)-CH_2_-C*H*_2_, CH(*i*Pr)-C*H*_2_-CH_2_, C*H*(*i*Pr)-CH_2_-CH_2_), 2.03–2.19 (m, 1H, CH(*i*Pr)-CH_2_-C*H*_2_), 4.63 (s, 1H, *trans*, C*H*_2_=C-CH=C), 4.74 (s, 1H, *cis*, C*H*_2_=C-CH=C), 5.85 (s, 1H, CH_2_=C-C*H*=C). ^13^C NMR (CDCl_3_, r.t.): δ 19.9 (cy-CH(*C*H_3_)_2_), 21.9 (cy-CH(*C*H_3_)_2_), 23.6 (CH_2_=C-CH=C-*C*H_3_), 24.7 (CH(*i*Pr)-*C*H_2_-CH_2_), 27.0 (cy-*C*H(CH_3_)_2_), 27.7 (CH(*i*Pr)-CH_2_-*C*H_2_), 46.2 (*C*H(*i*Pr)-CH_2_-CH_2_), 109.4 (*C*H_2_=C-CH=C), 125.1 (CH_2_=C-*C*H=C), 137.6 (CH_2_=C-CH=*C*), 146.4 (CH_2_=*C*-CH=C). ^1^H, ^13^C, COSY, and HMQC NMR spectra are shown in Figures S11, S14, and S15.

### 3.5. RAFT Copolymerization

RAFT copolymerization was carried out using the syringe technique under dry nitrogen in sealed glass tubes. A typical example for (–)-HCvD and MA copolymerization with CBTC and VAm-110 in toluene is given below. (−)-HCvD (0.60 mL, 3.3 mmol), MA (0.30 mL, 3.3 mmol), CBTC (0.13 mL, 508 mM solution in toluene, 0.066 mmol), and VAm-110 (1.19 mL of 18.6 mM solution in toluene, 0.022 mmol) were placed in a baked 25 mL graduated Schlenk flask equipped with a three-way stopcock at room temperature. The total volume of the reaction mixture was 2.2 mL. Immediately after mixing, aliquots (0.4 mL each) of the solution were distributed via syringe into baked glass tubes, which were then sealed by flame in a nitrogen atmosphere. The tubes were immersed in a thermostatic oil bath at 100 °C. At predetermined intervals, the polymerization was terminated by cooling the reaction mixtures to −78 °C. The monomer conversion was determined from the concentration of residual monomer measured by ^1^H NMR with toluene as an internal standard (20 h, 32% for (−)-HCvD and 26% for MA). The quenched reaction solutions were evaporated to dryness to give the product copolymer (*M*_n_ = 3100, *M*_w_/*M*_n_ = 1.24).

### 3.6. Measurements

The ^1^H NMR spectra for the monomer conversion and product polymer were recorded on a JEOL ECS-400 spectrometer operating at 400 MHz. The number-average molecular weight (*M*_n_) and molecular weight distribution (*M*_w_/*M*_n_) of the product polymer were determined by SEC in THF at 40 °C on two polystyrene gel columns (Tosoh Multipore H_xL_-M (7.8 mm i.d. × 30 cm) × 2; flow rate: 1.0 mL/min) connected to a JASCO PU-2080 precision pump and a JASCO RI-2031 detector. The columns were calibrated with standard polystyrene samples (Agilent Technologies; *M*_p_ = 580-3,242,000, *M*_w_/*M*_n_ = 1.02–1.23). The glass transition temperature (*T*_g_) of the polymers was recorded on a Q200 differential scanning calorimeter (TA Instruments, Inc.). Samples were first heated to 150 °C at 10 °C/min, equilibrated at this temperature for 10 min, and cooled to 0 °C at 5 °C/min. After being held at this temperature for 5 min, the samples were then reheated to 200 °C at 10 °C/min. All *T*_g_ values were obtained from the second scan after removing the thermal history.

## 4. Conclusions

In conclusion, radical polymerizations of a series of terpenoid-derived *exo*-methylene 6-membered ring conjugated dienes were investigated with the aim of developing novel biobased polymeric materials. Although their reactivities for homopolymerization in radical processes were lower than those in cationic processes, copolymerizations with various common vinyl monomers efficiently occurred, resulting in unique biobased copolymers. The substituents on the 6-membered ring of *exo*-methylene conjugated dienes affected the regiospecificity and monomer reactivity. The obtained copolymers contained relatively high biobased units, ranging from 40 to 60 mol% even at a 1:1 feed ratio, and the glass transition temperatures were comparable to those of the homopolymers obtained from common vinyl monomers. In addition, biobased units can be incorporated into copolymers in a controlled fashion via the RAFT process. We believe that these *exo*-methylene-conjugated dienes can be new building blocks to synthesize copolymers with terpenoid-derived alicyclic structures, which serve as hydrocarbon units with good thermal properties.

## Figures and Tables

**Table 1:** Radical polymerization of a series of terpenoid-derived *exo*-methylene 6-membered ring conjugated dienes *^a^*.

Entry	Monomer	Temp. (°C)	Conv. (%) *^b^*	*M*_n_(SEC) *^c^*	*M*_w_/*M*_n_*^c^*	1,4-/1,2- *^d^*	*T*_g_ (°C) *^e^*
1	β-Phe	60	2	3700	2.82	93/7	n.d. *^f^*
2	β-Phe	100	27	17,700	1.79	83/17	66
3	HCvD	60	0	10,800	3.03	>99/0	n.d. *^f^*
4	HCvD	100	32	34,400	1.89	>99/0	105
5	PtD	60	0	3200	2.47	>99/0	n.d. *^f^*
6	PtD	100	29	10,700	1.69	>99/0	113
7	VnD	60	0	240	1.10	n.d.	n.d. *^f^*
8	VnD	100	2	300	1.15	n.d.	n.d. *^f^*

*^a^* Polymerization condition: [M]_0_/[radical initiator]_0_ = 5000/30 mM in toluene at 60 (AIBN) or 100 °C (VAm-110), reaction time = 280 h. *^b^* Determined by ^1^H NMR of reaction mixture. *^c^* Determined by size-exclusion chromatography (SEC). *^d^* Determined by ^1^H NMR of the obtained polymers. *^e^* Determined by DSC. *^f^* Not determined.

**Table 2:** Radical copolymerization of terpenoid-derived *exo*-methylene 6-membered ring conjugated dienes (M_1_) and various common vinyl monomers (M_2_) in toluene at 100 °C *^a^*.

Entry	M_1_	M_2_	Time (h)	Conv.(%) *^b^* M_1_/M_2_	*M*_n_ (SEC) *^c^*	1,4-/1,2- *^d^*	M_1_/M_2_ (NMR) *^d^*	M_1_/M_2_ (Calcd) *^e^*	*r*_1_ *^f^*	*r*_2_ *^f^*
1	β-Phe	MA	90	38/27	7900	83/17	61/39	58/42	0.66	0.04
2	HCvD	MA	20	44/36	26,000	95/5	52/48	55/45	0.14	0.08
3	PtD	MA	20	43/35	23,800	96/4	51/49	55/45	0.11	0.08
4	β-Phe	AN	40	51/45	14,600	84/16	52/48	53/47	0.17	0.03
5	HCvD	AN	10	44/50	42,100	95/5	53/47	47/53	0.09	0.03
6	PtD	AN	10	45/50	42,300	99/1	50/50	47/53	0.02	0.05
7	β-Phe	MMA	150	36/31	7800	89/11	60/40	54/46	0.68	0.09
8	HCvD	MMA	50	44/51	12,900	95/5	47/53	46/54	0.17	0.31
9	PtD	MMA	120	55/62	13,500	>99/0	46/54	47/53	0.19	0.25
10	β-Phe	St	120	38/29	6700	85/15	60/40	57/43	1.53	0.43
11	HCvD	St	100	40/54	19,000	95/5	43/57	43/57	0.40	1.09
12	PtD	St	50	29/35	10,900	>99/0	41/59	45/55	0.48	0.96

*^a^* Polymerization condition: [M_1_]_0_/[M_2_]_0_/[VAm-110]_0_ = 1500/1500/30 mM in toluene at 100 °C. *^b^* Determined by ^1^H NMR of reaction mixture. *^c^* Determined by SEC. *^d^* Determined by ^1^H NMR of the obtained polymers. *^e^* Determined by the monomer feed ratio and monomer conversion. *^f^* Determined by Kelen–Tüdõs method for the copolymerizations at varying monomer feed ratios.

## Supplementary Materials

The following are available online Figure S1. SEC curves of the homopolymers obtained in the radical polymerization of β-Phe, (–)-HCvD, and PtD: [M]_0_/[VAm-110]_0_ = 5000/30 mM in toluene at 100 °C; Figure S2. ^13^C NMR spectra (in C_2_D_2_Cl_4_ at 100 °C) of poly((–)-HCvD) obtained in the cationic (A) and radical (B) polymerization: [(–)-HCvD]_0_/[CEVE-HCl]_0_/[SnCl_4_]_0_/[*n*Bu_4_NCl]_0_ = 100/1.0/5.0/4.0 mM in toluene/CH_2_Cl_2_ (50/50 vol%) at –78 °C (*M*_n_(Calcd) = 15,200) (A) or [(–)-HCvD]_0_/[VAm-110]_0_ = 5000/30 mM in toluene at 100 °C (B); Figure S3. Differential scanning calorimetry (DSC) curves of poly(β-Phe), poly((–)-HCvD), and poly(PtD) obtained in the radical polymerization: [M]_0_/[VAm-110]_0_ = 5000/30 mM in toluene at 100 °C; Figure S4. Time-conversion curves for the radical copolymerization of terpenoid-derived *exo*-methylene 6-membered ring conjugated dienes with MA (A), AN (B), MMA (C), and St (D) as a comonomer: [diene]_0_/[comonomer]_0_/[AIBN]_0_ = 1500/1500/30 mM in toluene at 60 °C; Figure S5. SEC curves of the copolymers obtained in the radical copolymerization of terpenoid-derived *exo*-methylene 6-membered-ring conjugated dienes (M_1_) with various common vinyl monomers (M_2_): [M_1_]_0_/[M_2_]_0_/[VAm-110]_0_ = 1500/1500/30 mM in toluene at 100 °C; Figure S6. ^1^H NMR spectra (in CDCl_3_ at 55 °C) of copolymers obtained in the radical copolymerization of β-Phe (A), (–)-HCvD (B), or PtD (C) with AN: [diene]_0_/[AN]_0_/[VAm-110]_0_ = 1500/1500/30 mM in toluene at 100 °C; Figure S7. ^1^H NMR spectra (in CDCl_3_ at 55 °C) of copolymers obtained in the radical copolymerization of β-Phe (A), (–)-HCvD (B), or PtD (C) with MMA: [diene]_0_/[MMA]_0_/[VAm-110]_0_ = 1500/1500/30 mM in toluene at 100 °C; Figure S8. ^1^H NMR spectra (in CDCl_3_ at 55 °C) of copolymers obtained in the radical copolymerization of β-Phe (A), (–)-HCvD (B), or PtD (C) with St: [diene]_0_/[St]_0_/[VAm-110]_0_ = 1500/1500/30 mM in toluene at 100 °C; Figure S9. Time-conversion curves for the RAFT copolymerization of (–)-HCvD with MA (A), AN (B), MMA (C), and St (D) as a comonomer: [(–)-HCvD]_0_/[comonomer]_0_/[CBTC]_0_/[VAm-110]_0_ = 1500/1500/30/10 mM in toluene at 100 °C; Figure S10. ^1^H NMR spectra (in CDCl_3_ at 55 °C) of copolymers obtained in the RAFT copolymerization of (–)-HCvD with MA (A), AN (B), MMA (C), or St (D) as a comonomer: [(–)-HCvD]_0_/[comonomer]_0_/[CBTC]_0_/[VAm-110]_0_ = 1500/1500/30/10 mM in toluene at 100 °C; Figure S11. ^1^H NMR spectra of (–)-VnD (A) and PtD (B) in CDCl_3_ at 25 °C; Figure S12. ^13^C NMR and DEPT spectra of (–)-VnD in CDCl_3_ at 25 °C; Figure S13. ^1^H-^1^H COSY and HMQC spectra of (–)-VnD in CDCl_3_ at 25 °C; Figure S14. ^13^C NMR and DEPT spectra of PtD in CDCl_3_ at 25 °C; Figure S15. ^1^H-^1^H COSY and HMQC spectra of PtD in CDCl_3_ at 25 °C; Table S1. Radical copolymerization of terpenoid-derived exo-methylene 6-membered ring conjugated dienes (M_1_) and various common vinyl monomers (M_2_) in toluene at 60 °C.
