# Supplementary material for: Biobased Polymers via Radical Homopolymerization and Copolymerization of a Series of Terpenoid-Derived Conjugated Dienes with exo-Methylene and 6-Membered Ring
Source: Molecules. 2020 Dec 12;25(24):5890. doi: 10.3390/molecules25245890 (PMC7763260; doi:10.3390/molecules25245890)
Supplement: Supplementary file 1 [file molecules-25-05890-s001.pdf]

Supporting Information

Biobased Polymers via Radical Homopolymerization and Copolymerization of a Series of Terpenoid-Derived Conjugated Dienes with *exo*-Methylene and 6-Membered Ring

Takenori Nishida,[†] Kotaro Satoh,[†,‡] and Masami Kamigaito[*,†]

[†]*Department of Molecular and Macromolecular Chemistry, Graduate School of Engineering, Nagoya University, Furo-cho, Chikusa-ku, Nagoya 464-8603, Japan*

[‡]*Department of Chemical Science and Engineering, School of Materials and Chemical Technology, Tokyo Institute of Technology, 2-12-1-H120 Ookayama, Meguro-ku, Tokyo 152-8550, Japan*

e-mail: kamigait@chembio.nagoya-u.ac.jp

Contents:

Figure S1 ········ S2
Figure S2 ········ S2
Figure S3 ········ S3
Figure S4 ········ S3
Table S1 ········ S4
Figure S5 ········ S4
Figure S6 ········ S5
Figure S7 ········ S6
Figure S8 ········ S7
Figure S9 ········ S8
Figure S10 ········ S9
Figure S11 ········ S10
Figure S12 ········ S11
Figure S13 ········ S12
Figure S14 ········ S13
Figure S15 ········ S14

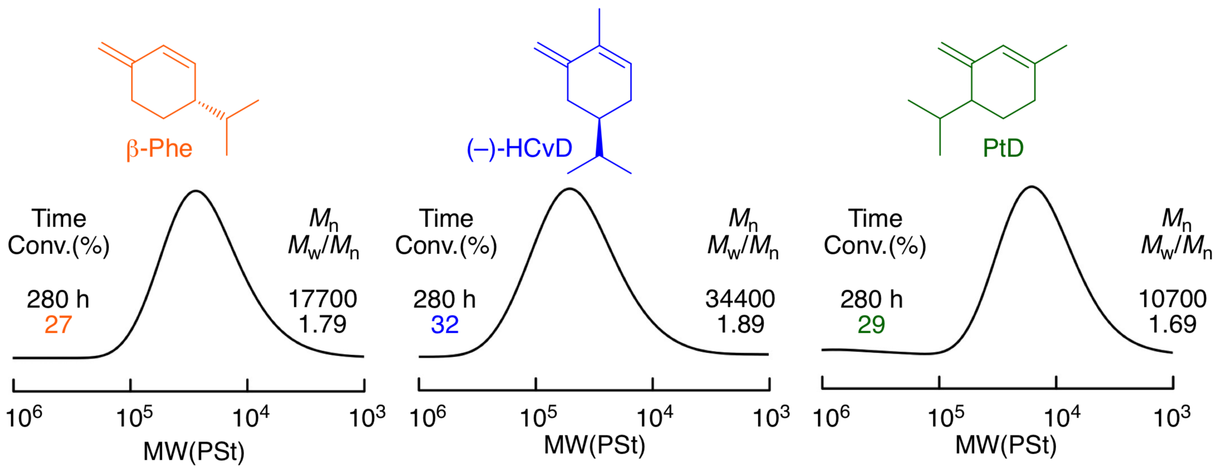

Figure S1. SEC curves of the homopolymers obtained in the radical polymerization of β-Phe, (–)-HCvD, and PtD: $[M]_0/[VAm\text{-}110]_0$ = 5000/30 mM in toluene at 100 °C.

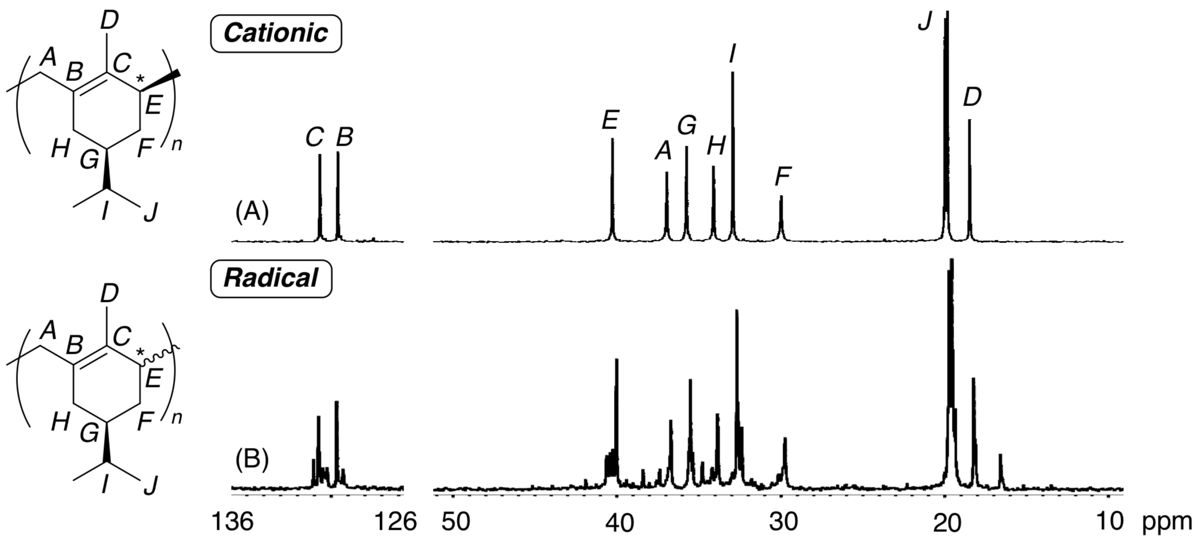

Figure S2. ^{13}C NMR spectra (in $C_2D_2Cl_4$ at 100 °C) of poly((–)-HCvD) obtained in the cationic (A) and radical (B) polymerization: $[(–)\text{-HCvD}]_0/[\text{CEVE-HCl}]_0/[SnCl_4]_0/[n\text{Bu}_4\text{NCl}]_0$ = 100/1.0/5.0/4.0 mM in toluene/CH_2Cl_2 (50/50 vol%) at –78 °C (M_n(Calcd) = 15200) (A) or $[(–)\text{-HCvD}]_0/[VAm\text{-}110]_0$ = 5000/30 mM in toluene at 100 °C (B).

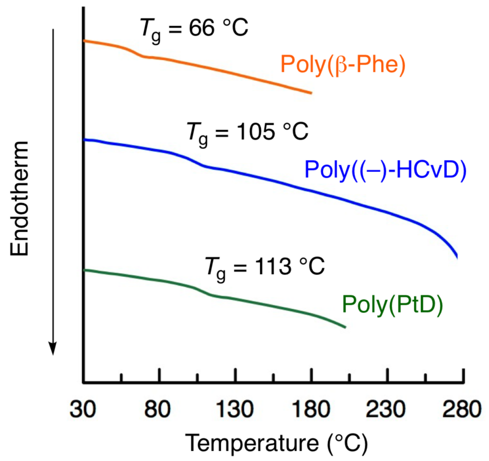

Figure S3. Differential scanning calorimetry (DSC) curves of poly(β-Phe), poly((−)-HCvD), and poly(PtD) obtained in the radical polymerization: $[M]_0/[VAm\text{-}110]_0$ = 5000/30 mM in toluene at 100 °C.

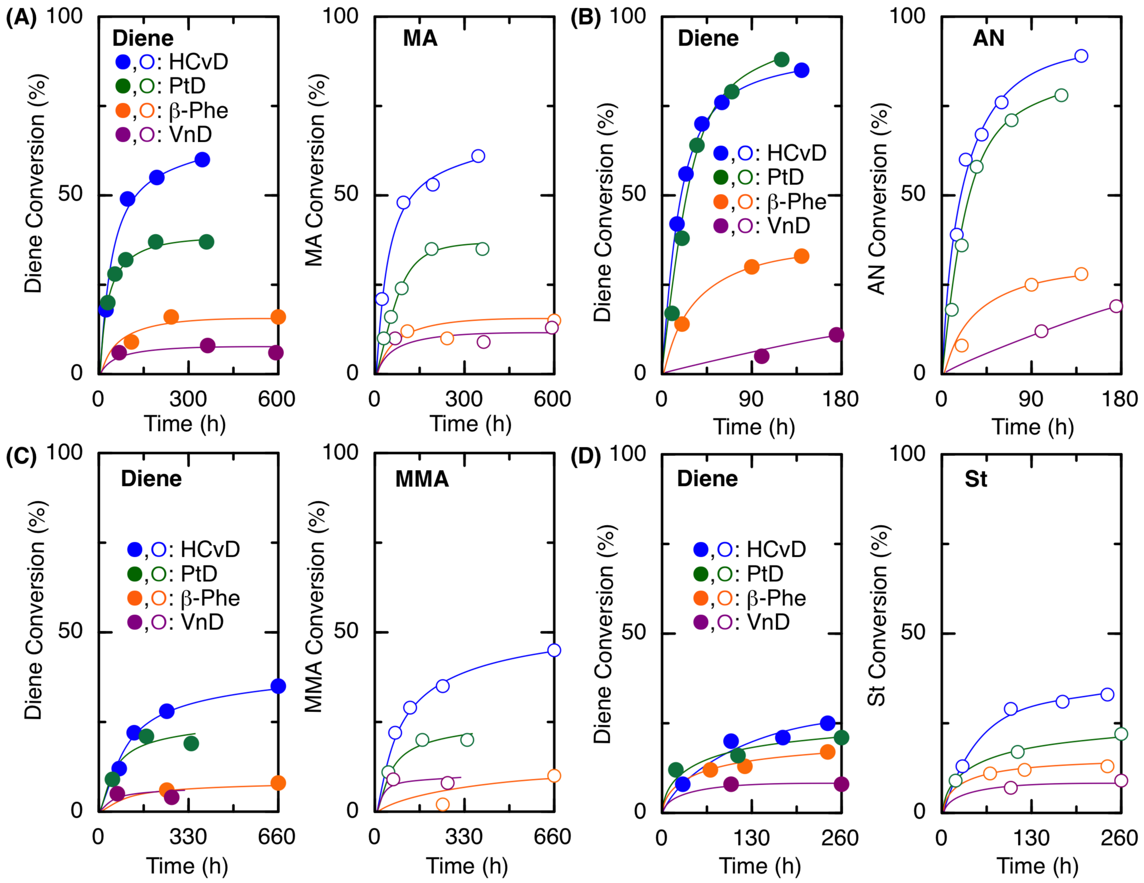

Figure S4. Time-conversion curves for the radical copolymerization of terpenoid-derived *exo*-methylene 6-membered ring conjugated dienes with MA (A), AN (B), MMA (C), and St (D) as a comonomer: $[\text{diene}]_0/[\text{comonomer}]_0/[\text{AIBN}]_0$ = 1500/1500/30 mM in toluene at 60 °C.

Table S1. Radical copolymerization of terpenoid-derived *exo*-methylene 6-membered ring conjugated dienes (M_1) and various common vinyl monomers (M_2) in toluene at 60 °C[a].

Entry	M_1	M_2	Time (h)	Conv. (%)[b] M_1/M_2	M_n(SEC)[c]	M_w/M_n[c]
1	β-Phe	MA	242	17/11	1600	2.80
2	HCvD	MA	96	49/48	11700	1.62
3	PtD	MA	90	32/25	6300	1.68
4	VnD	MA	364	8/9	480	1.87
5	β-Phe	AN	90	30/25	4700	1.96
6	HCvD	AN	24	56/60	19400	1.68
7	PtD	AN	35	64/58	14700	1.61
8	VnD	AN	175	11/19	1100	1.65
9	β-Phe	MMA	130	6/2	1700	2.60
10	HCvD	MMA	340	22/29	7900	1.91
11	PtD	MMA	250	19/20	4500	2.49
12	VnD	MMA	268	4/8	350	1.80
13	β-Phe	St	240	17/13	1400	2.51
14	HCvD	St	175	21/27	6600	2.20
15	PtD	St	110	16/17	2400	1.87
16	VnD	St	260	8/9	460	1.36

[a]Polymerization condition: $[M_1]_0/[M_2]_0/[AIBN]_0$ = 1500/1500/30 mM in toluene at 60 °C. [b]Determined by ^{1}H NMR of reaction mixture. [c]Determined by SEC.

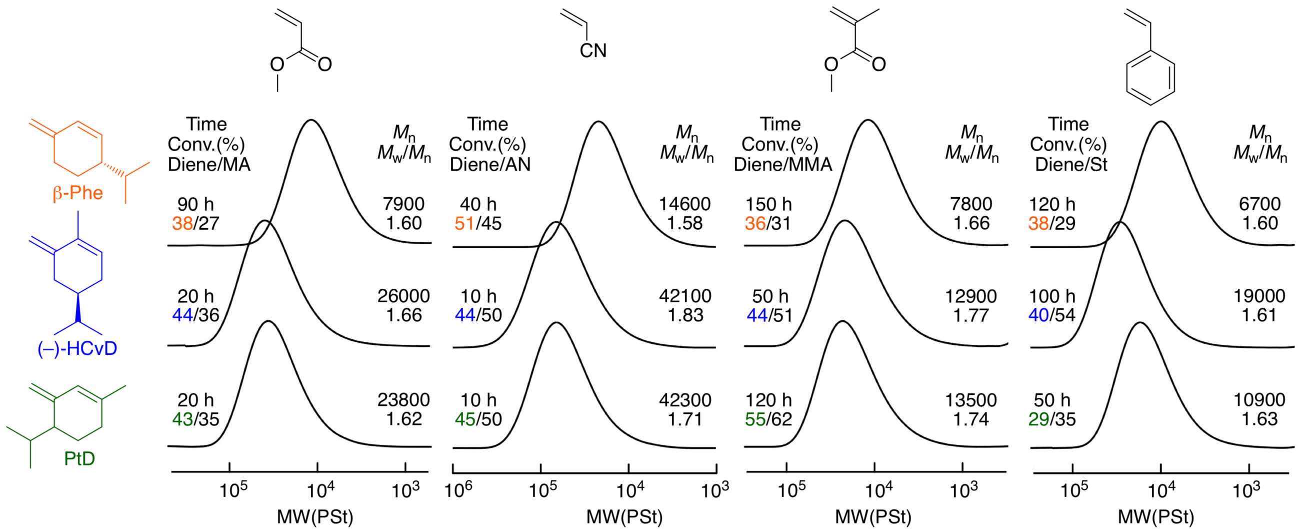

Figure S5. SEC curves of the copolymers obtained in the radical copolymerization of terpenoid-derived *exo*-methylene 6-membered-ring conjugated dienes (M_1) with various common vinyl monomers (M_2): $[M_1]_0/[M_2]_0/[VAm\text{-}110]_0$ = 1500/1500/30 mM in toluene at 100 °C.

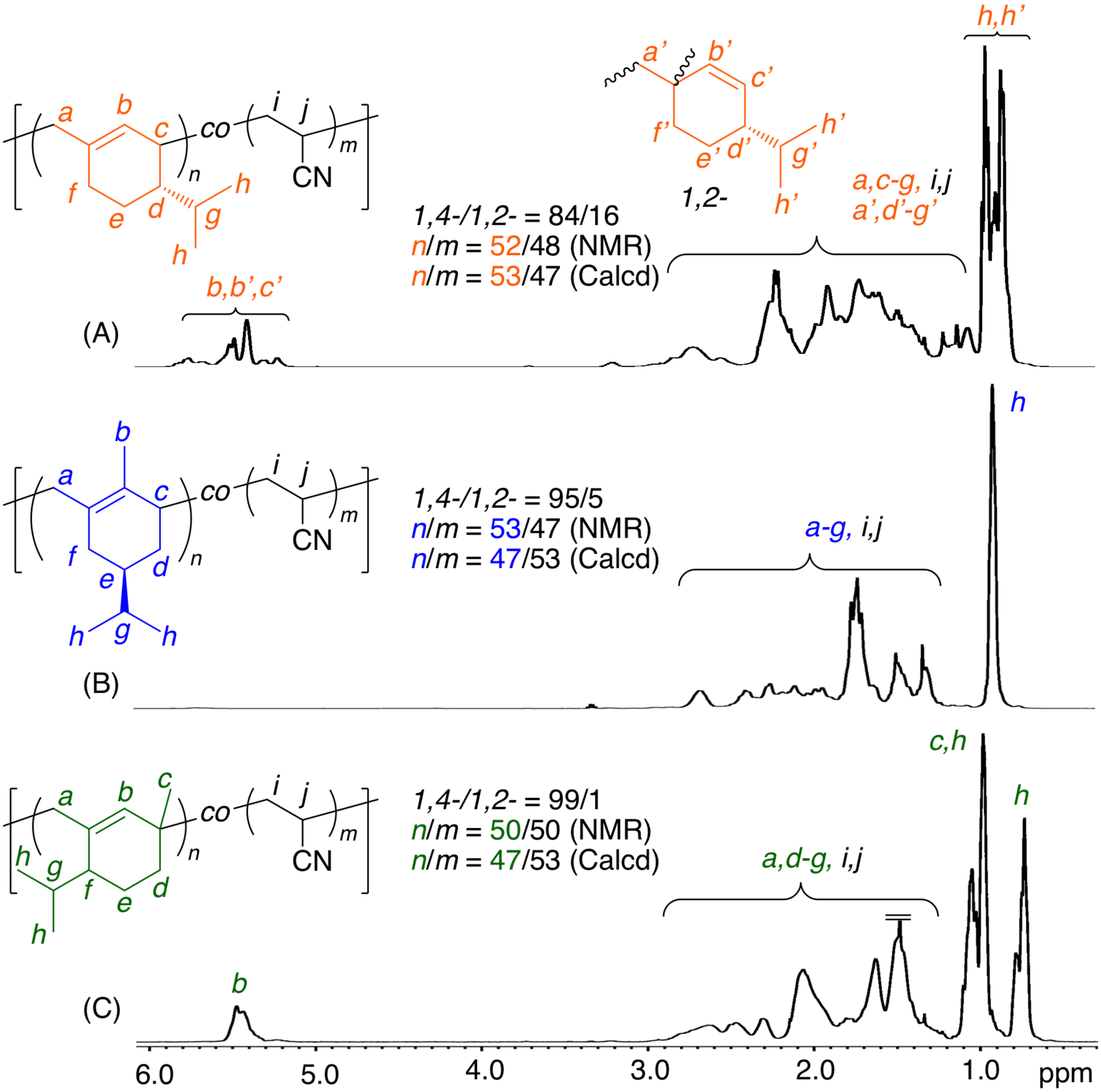

Figure S6. ^{1}H NMR spectra (in $CDCl_3$ at 55 °C) of copolymers obtained in the radical copolymerization of β-Phe (A), (–)-HCvD (B), or PtD (C) with AN: $[\text{diene}]_0/[\text{AN}]_0/[\text{VAm-110}]_0$ = 1500/1500/30 mM in toluene at 100 °C.

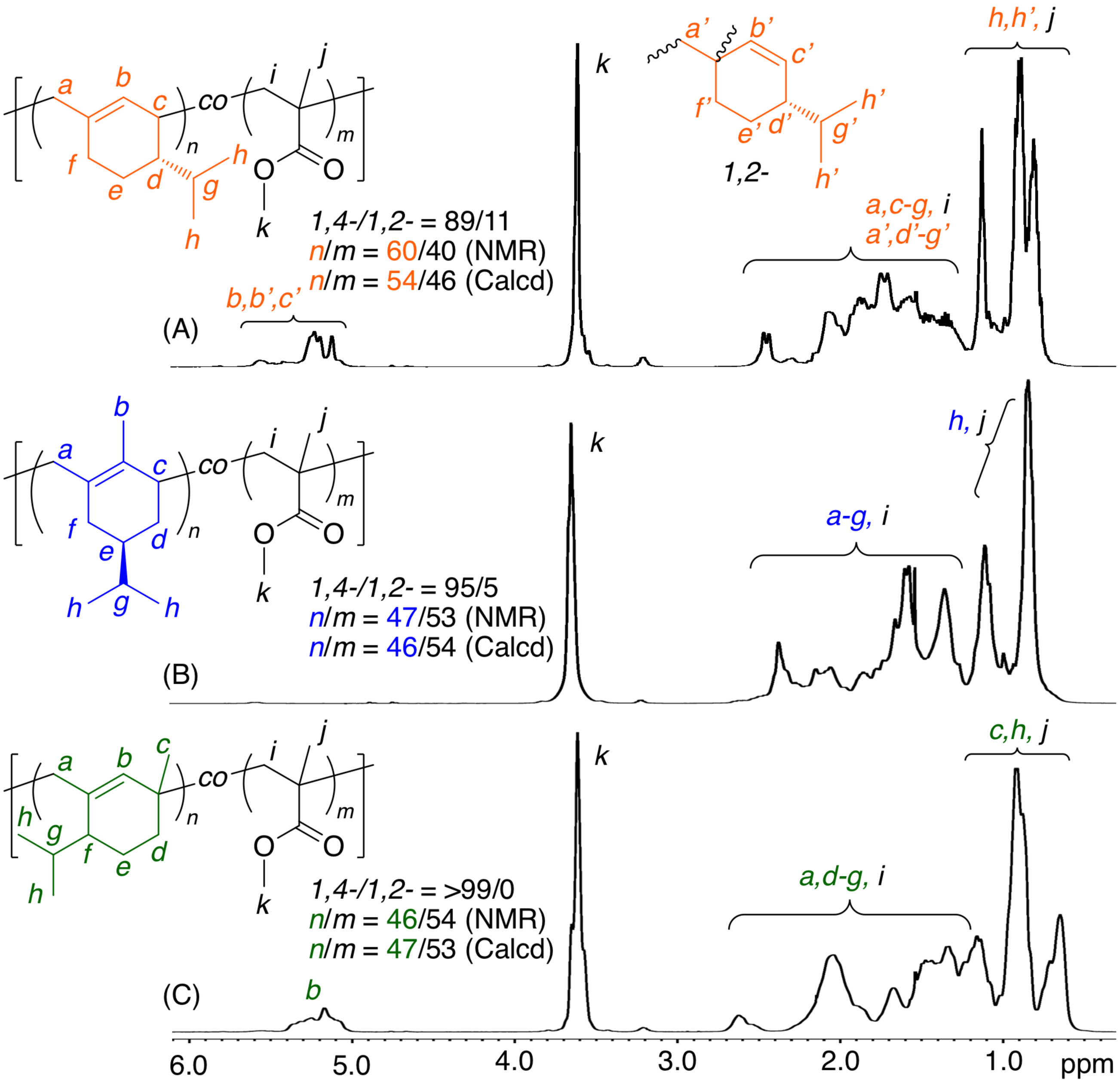

Figure S7. ^{1}H NMR spectra (in $CDCl_3$ at 55 °C) of copolymers obtained in the radical copolymerization of β-Phe (A), (–)-HCvD (B), or PtD (C) with MMA: $[diene]_0/[MMA]_0/[VAm\text{-}110]_0$ = 1500/1500/30 mM in toluene at 100 °C.

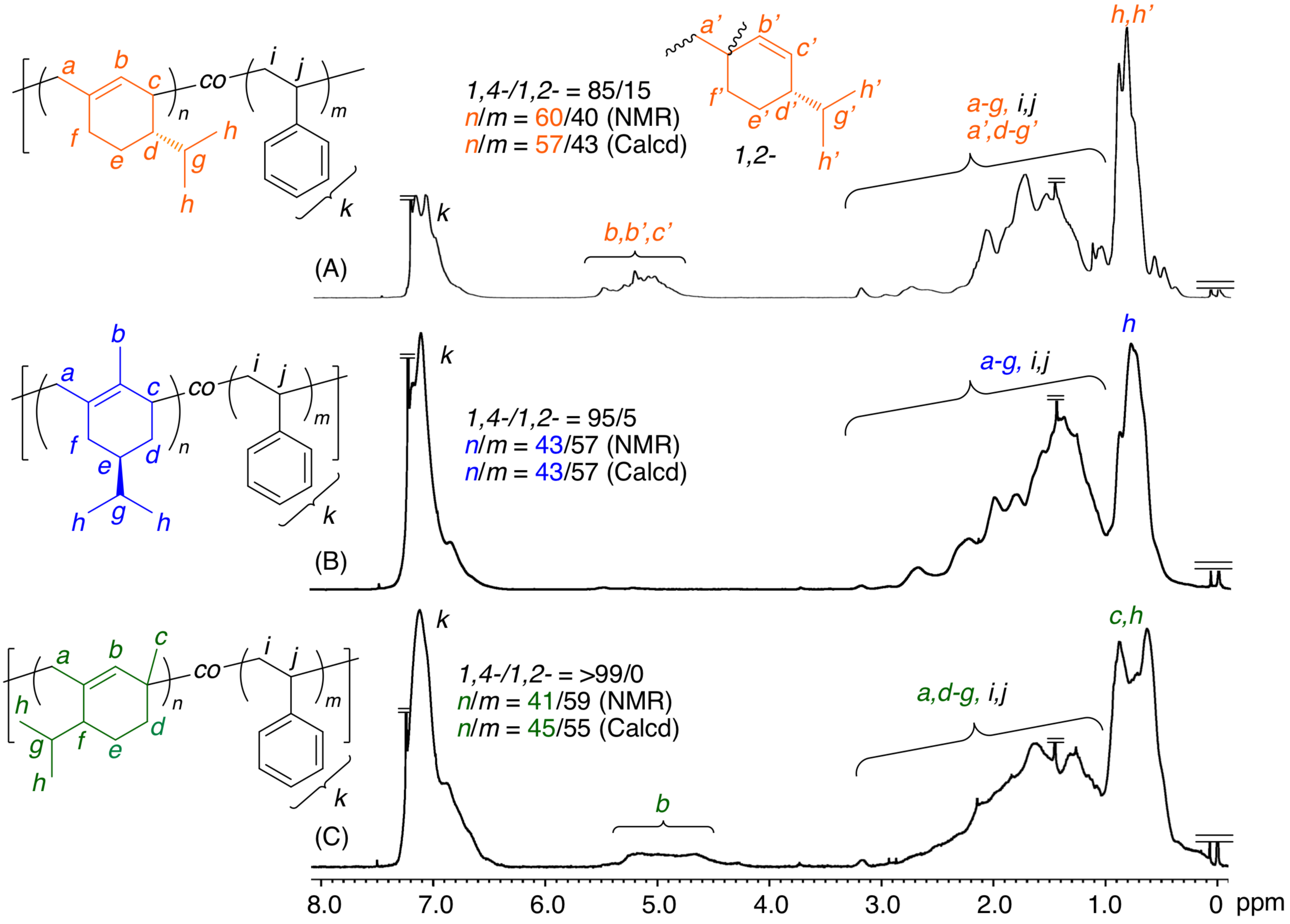

Figure S8. ^{1}H NMR spectra (in $CDCl_3$ at 55 °C) of copolymers obtained in the radical copolymerization of β-Phe (A), (–)-HCvD (B), or PtD (C) with St: $[diene]_0/[St]_0/[VAm\text{-}110]_0$ = 1500/1500/30 mM in toluene at 100 °C.

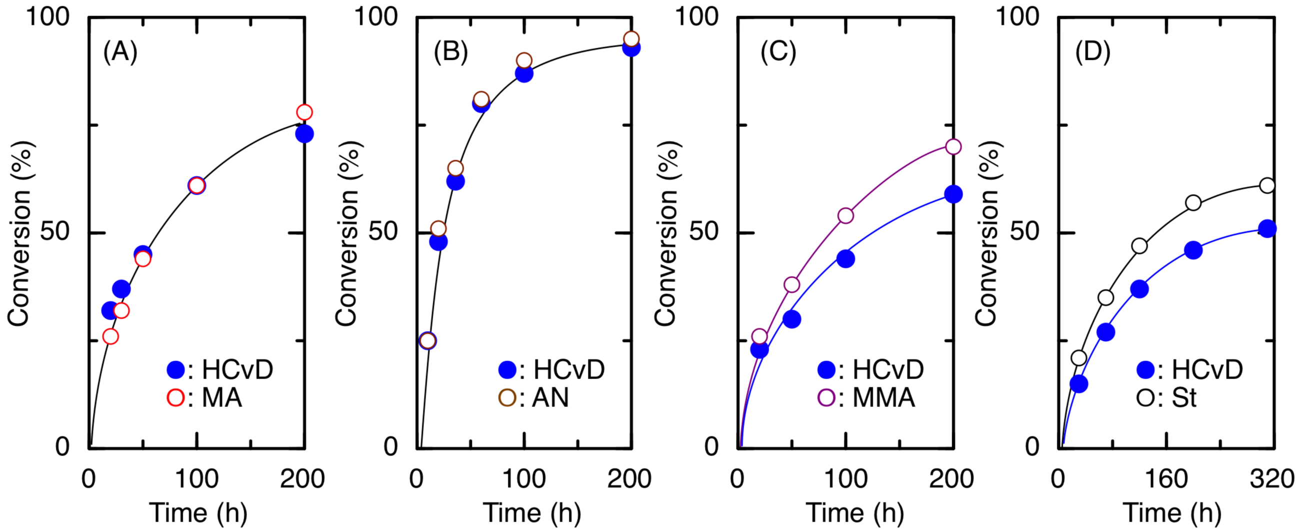

Figure S9. Time-conversion curves for the RAFT copolymerization of (–)-HCvD with MA (A), AN (B), MMA (C), and St (D) as a comonomer: $[(–)\text{-HCvD}]_0/[\text{comonomer}]_0/[\text{CBTC}]_0/[\text{VAm-110}]_0$ = 1500/1500/30/10 mM in toluene at 100 °C.

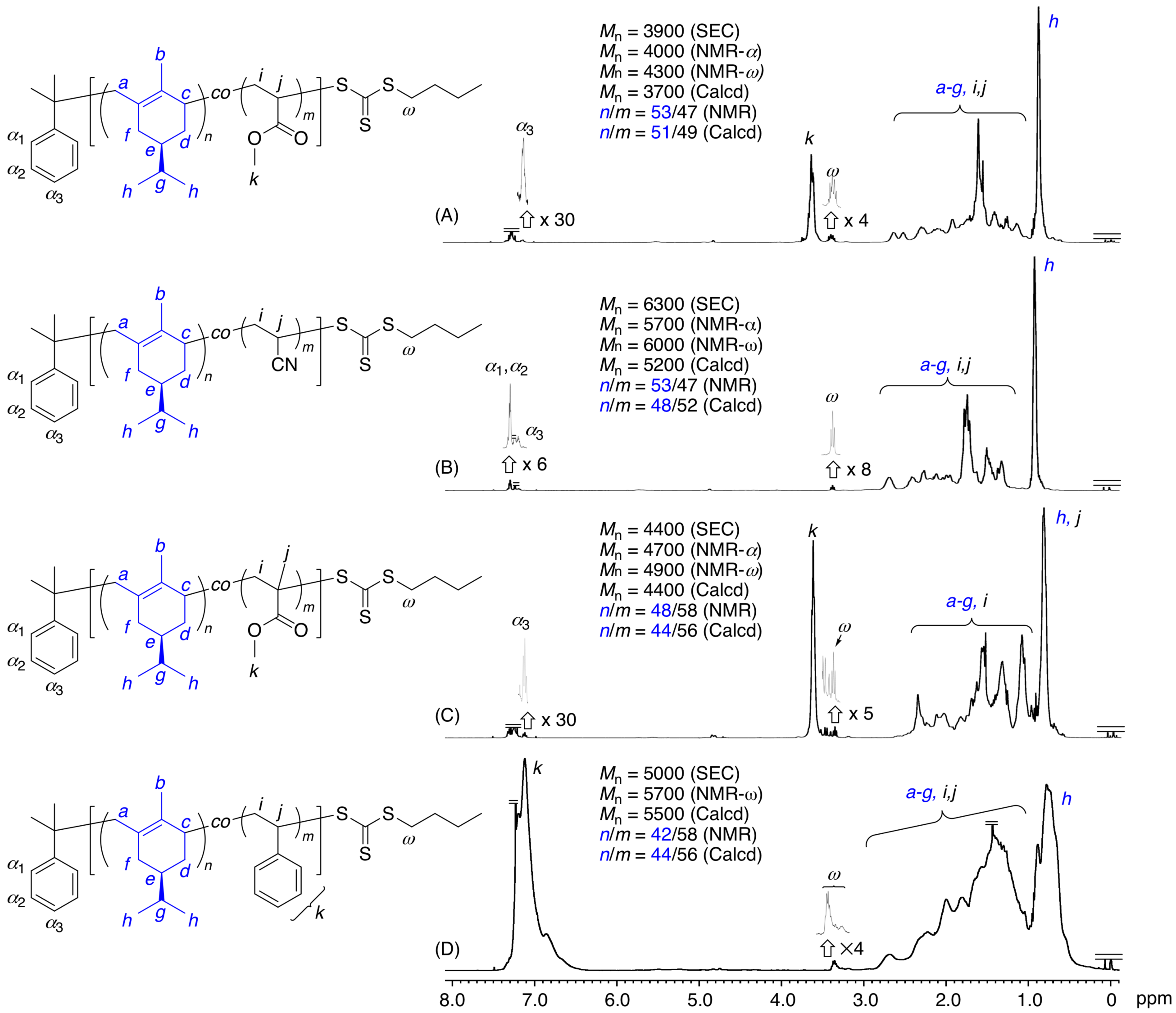

Figure S10. ^{1}H NMR spectra (in $CDCl_3$ at 55 °C) of copolymers obtained in the RAFT copolymerization of (–)-HCvD with MA (A), AN (B), MMA (C), or St (D) as a comonomer: $[(–)\text{-HCvD}]_0/[\text{comonomer}]_0/[\text{CBTC}]_0/[\text{VAm-110}]_0$ = 1500/1500/30/10 mM in toluene at 100 °C.

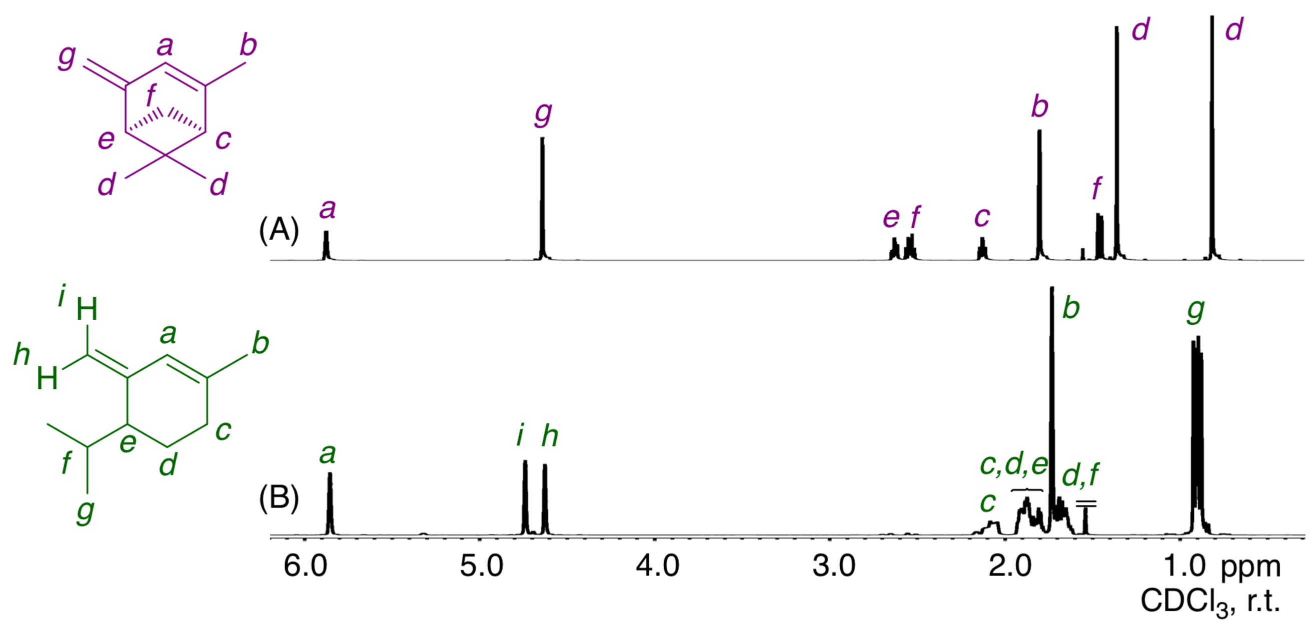

Figure S11. ^{1}H NMR spectra of (–)-VnD (A) and PtD (B) in $CDCl_3$ at 25 °C

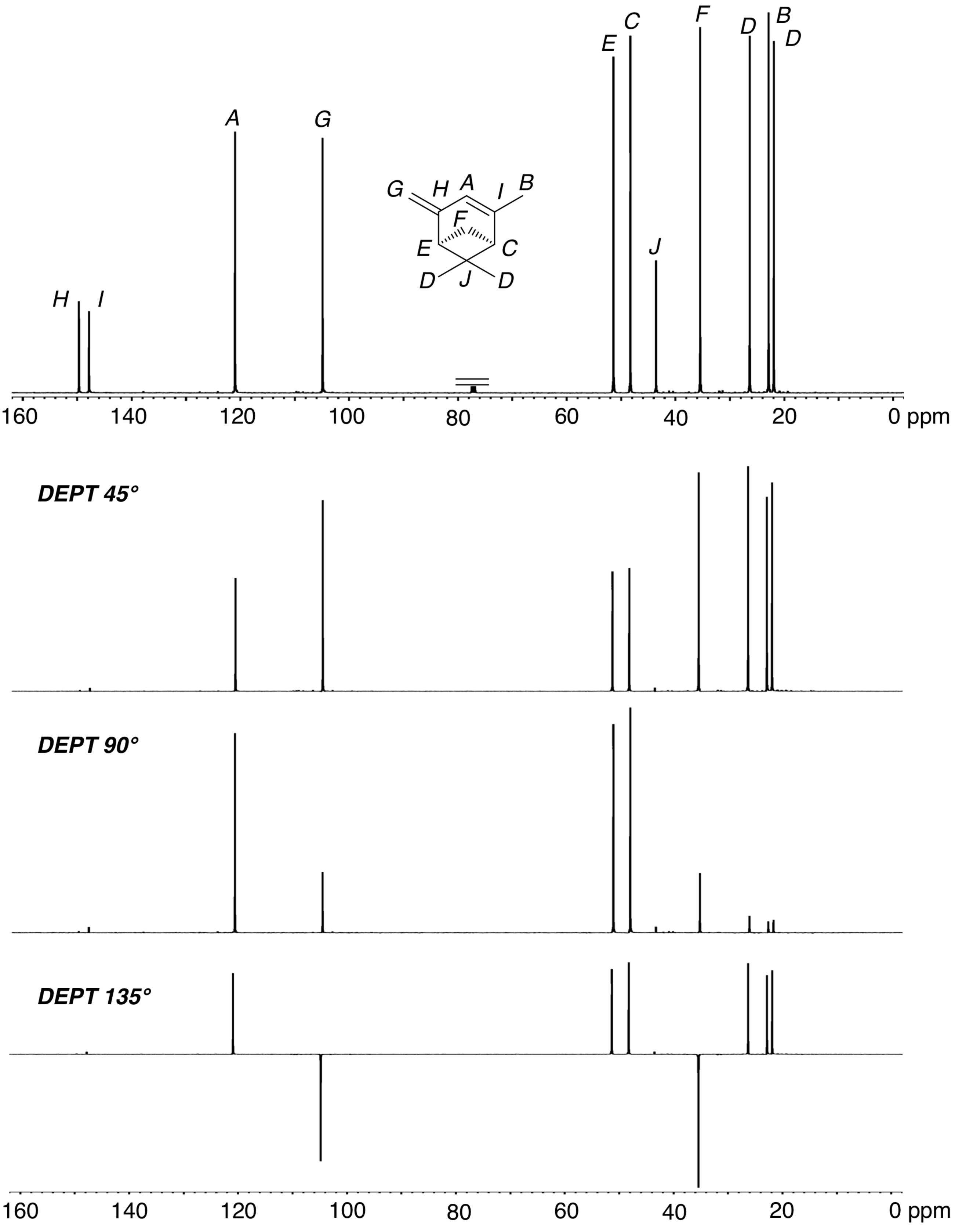

Figure S12. ^{13}C NMR and DEPT spectra of (–)-VnD in $CDCl_3$ at 25 °C.

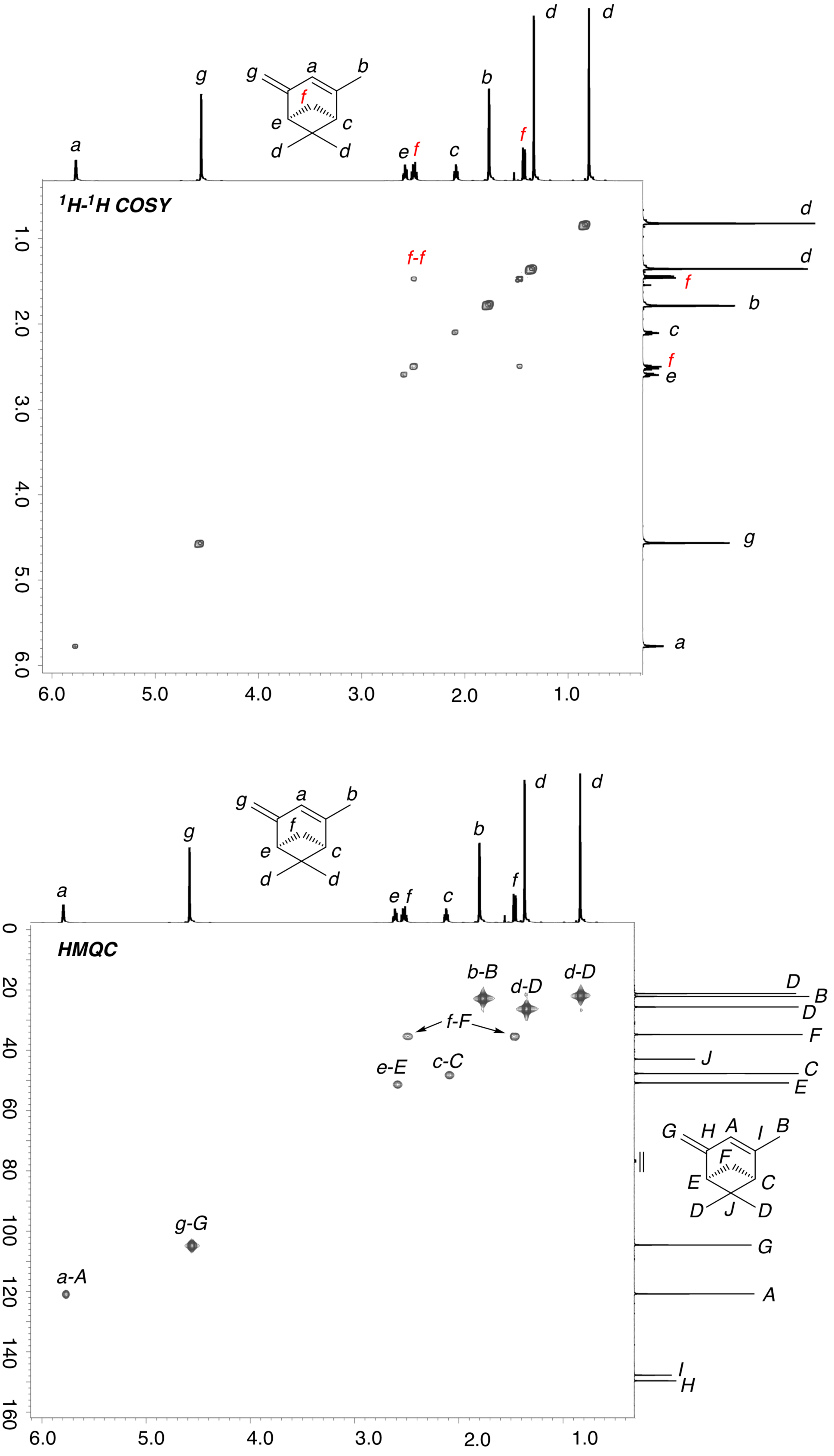

Figure S13. ^{1}H-^{1}H COSY and HMQC spectra of (–)-VnD in $CDCl_3$ at 25 °C.

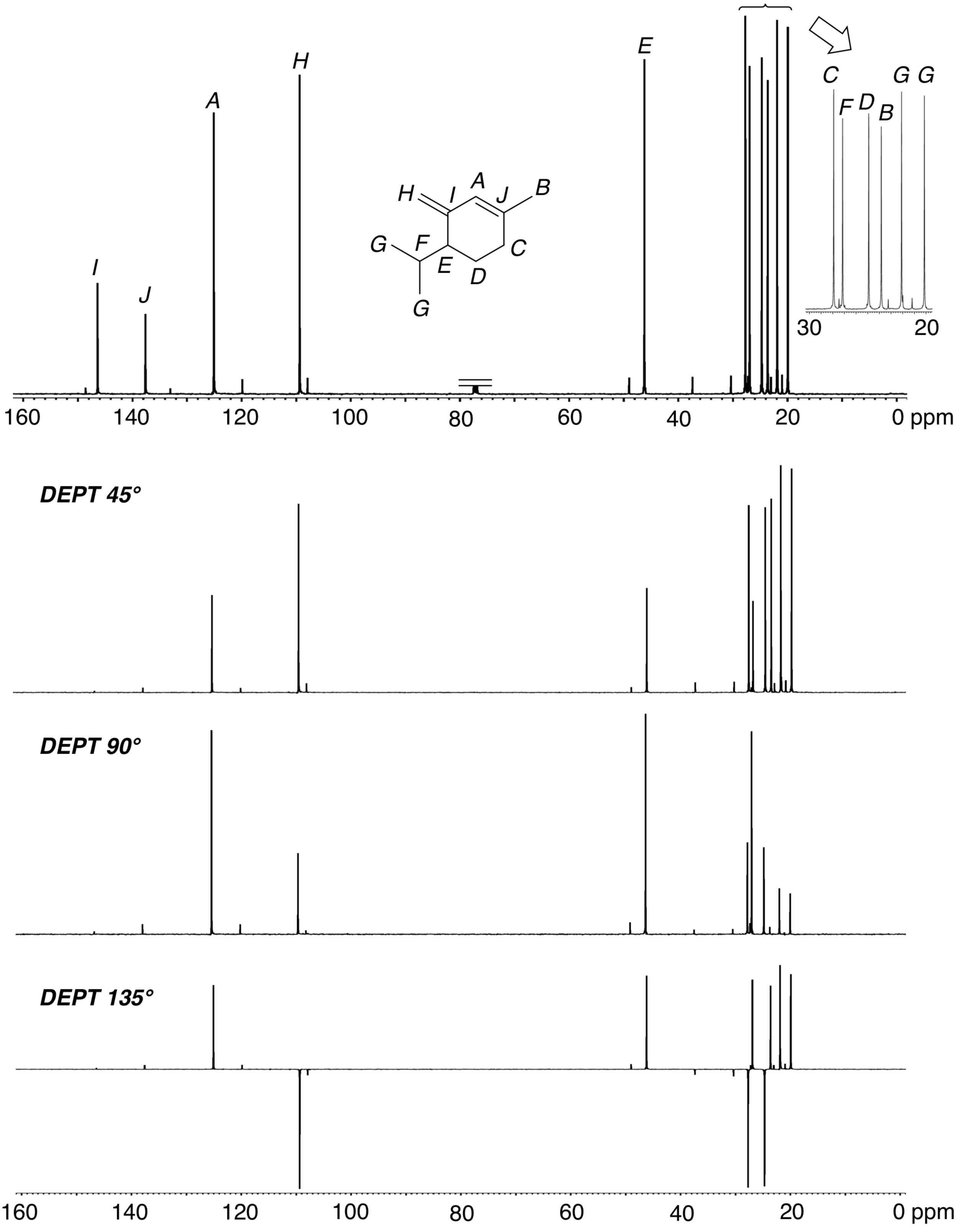

Figure S14. ^{13}C NMR and DEPT spectra of PtD in $CDCl_3$ at 25 °C.

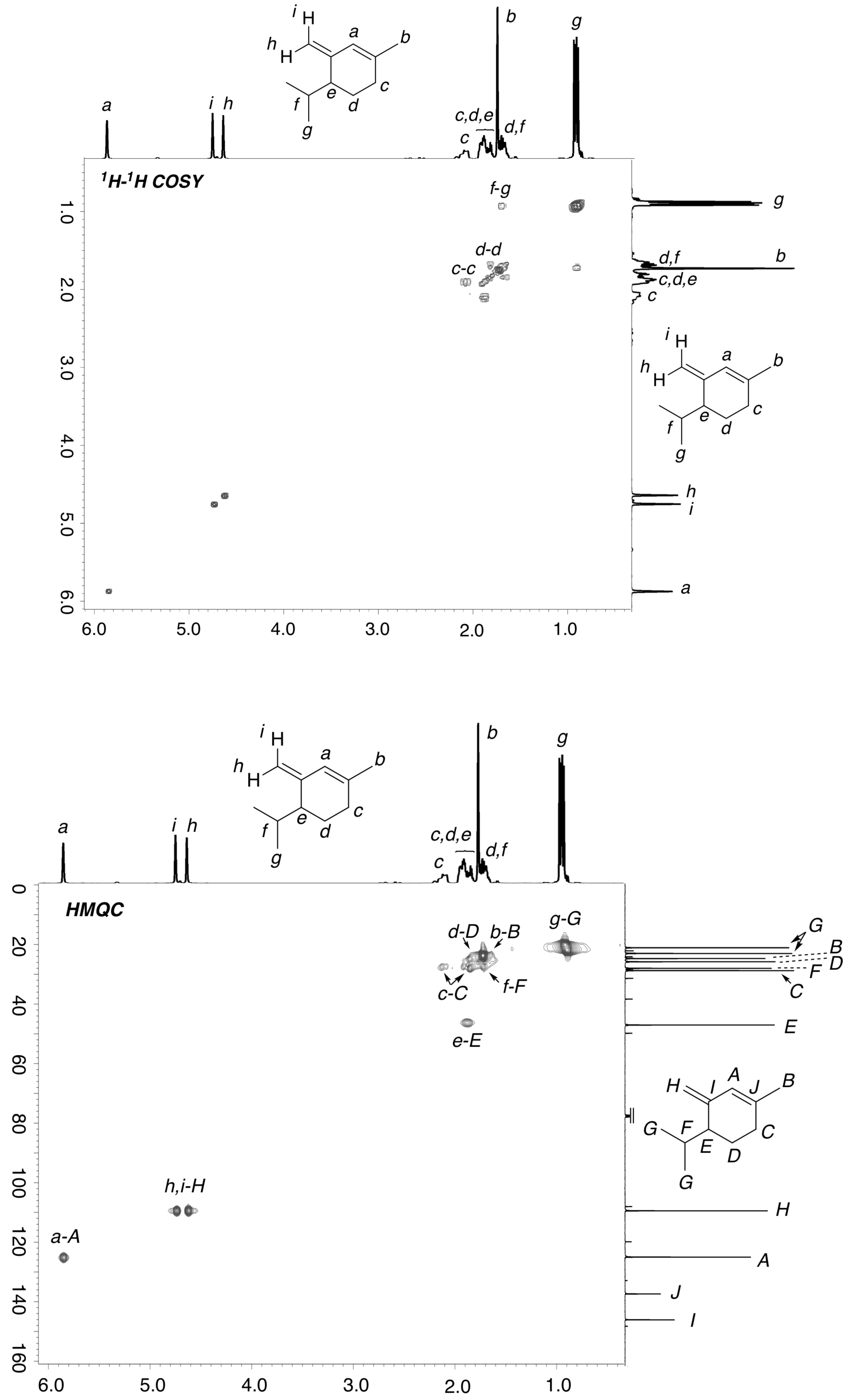

Figure S15. ^{1}H-^{1}H COSY and HMQC spectra of PtD in $CDCl_3$ at 25 °C.
